# Association of Independent Prognostic Factors and Treatment Modality With Survival and Recurrence Outcomes in Breast Cancer

**DOI:** 10.1001/jamanetworkopen.2020.7213

**Published:** 2020-07-09

**Authors:** Dat Nguyen, John Yu, William C. Reinhold, Sherry X. Yang

**Affiliations:** 1 National Clinical Target Validation Laboratory, Division of Cancer Treatment and Diagnosis, National Cancer Institute, Bethesda, Maryland; 2 DSC, Inc, Reston, Virginia; 3 currently affiliated with Bellese Technologies, LLC, Owings Mills, Maryland; 4 Laboratory of Pharmacology, Center for Cancer Research, National Cancer Institute, Bethesda, Maryland

## Abstract

**Importance:**

It is not well understood whether prognostic factors in breast cancer are affected by specific treatment and vary by clinical outcome type compared with untreated patients.

**Objective:**

To identify independent clinical and molecular measurements associated with overall survival (OS) and recurrence-free survival (RFS) by homogeneous treatment in women with breast cancer.

**Design, Setting, and Participants:**

This prognostic study included 956 patients diagnosed with invasive breast cancer from hospital centers across 4 geographical regions of the United States who participated in the accreditation program of the Commission on Cancer of the American College of Surgeons from 1985 to 1997. The duration of follow-up ranged from 1 to 282 months. The study analysis was conducted from June 10, 2019, to March 18, 2020.

**Main Outcomes and Measures:**

Analysis of OS and RFS in patients who underwent chemotherapy, radiotherapy, or endocrine therapy alone compared with no systemic or locoregional therapy. Cox proportional hazards regression models were used to estimate independent performance and 95% CI of age, tumor size, number of positive nodes (nodal status), tumor grades 2 and 3, p53 status, estrogen receptor (ER) status, and *ERBB2* (formerly *HER2*) status.

**Results:**

Among 956 participants, median age was 61 (range, 25-96) years. Age (adjusted hazard ratio [AHR], 2.24; 95% CI, 1.27-3.94; *P* = .01) and high grade (AHR, 2.05; 95% CI, 1.09-3.86; *P* = .02), in addition to nodal status and tumor size, were independently associated with OS and RFS, respectively, in untreated patients. p53 status (AHR, 2.11; 95% CI, 1.07-4.18; *P* = .03) and ER status (AHR, 0.46; 95% CI, 0.23-0.92; *P* = .03) were associated with higher and lower risks of death, respectively, whereas nodal status (AHR, 1.13; 95% CI, 1.06-1.20; *P* < .005), high grade (AHR, 4.01; 95% CI, 1.51-10.70; *P* = .01), and *ERBB2* positivity (AHR, 2.67; 95% CI, 1.25-5.70; *P* = .01) were associated with the risk of recurrence after endocrine therapy. Tumor size (AHR for OS, 2.76 [95% CI, 1.79-4.31; *P* < .005]; AHR for RFS, 2.27 [95% CI, 1.23-4.18; *P* = .01]) and *ERBB2* status (AHR for OS, 5.35 [95% CI, 1.31-21.98; *P* = .02]; AHR for RFS, 6.05 [95% CI, 1.48-24.78; *P* = .01]) were independently associated with radiotherapy outcomes, and nodal status was significantly associated with chemotherapy outcomes (AHR for OS, 1.06 [95% CI, 1.02-1.09; *P* < .005]; AHR for RFS, 1.05 [95% CI, 1.01-1.09; *P* = .01]).

**Conclusions and Relevance:**

In this study, independent prognostic factors were associated with specific treatment and weighted by the outcome category with reference to untreated patients within biological and clinical contexts.

## Key Points

**Question:**

Is the performance of clinical and molecular factors associated with distinct treatment and clinical outcome types in breast cancer?

**Findings:**

This prognostic study of 956 women with breast cancer analyzed overall and recurrence-free survival in patients undergoing homogeneous therapies and found a complete and partial deviation in the identification of independent prognostic factors from outcomes of untreated patients. Independent prognostic factors were differential in the context of endocrine therapy and largely concordant for radiotherapy and chemotherapy (but partly divergent from nontherapy) between survival and recurrence outcomes.

**Meaning:**

Performance of the independent clinical and molecular factors was weighted by treatment modality and the nature of clinical end points.

## Teaser

This prognostic study identifies independent clinical and molecular measurements associated with overall and recurrence-free survival by homogeneous treatment in patients with breast cancer.

## Introduction

Breast cancer is the most commonly diagnosed cancer and the second leading cause of cancer-related death for women in the United States. Current guidelines by the National Comprehensive Cancer Network and American Society of Clinical Oncology recommend management with endocrine therapy, *ERBB2-* (formerly *HER2*-) (OMIM 164870) directed therapy, radiotherapy, and cytotoxic chemotherapy or a combination for patients with invasive breast cancer.^1^ The choice of treatment modality depends on patient and tumor characteristics, expression of hormone receptors (HR; including estrogen receptor α [ER] or progesterone receptor [PR]), and *ERBB2* status as well as genomic test results such as the Oncotype DX breast recurrence score.^2^ Endocrine therapy with a duration of 5 to 10 years is a standard of care for HR-positive disease, which accounts for approximately 70% of all breast cancers.^3,4,5^ Radiotherapy applies to all individuals who underwent breast-conserving surgery and may be used for patients with a tumor larger than 5 cm or with node-positive disease after mastectomy. Chemotherapy is recommended for patients with *ERBB2*-positive and HR-negative tumors, node-positive disease, and high Oncotype recurrence scores in HR-positive and *ERBB2*-negative breast cancer.^3,6^

p53 is a nuclear transcription factor encoded by the *TP53* gene (OMIM 191170) located in the short arm of chromosome 17 (17p13.1). It regulates cell fate in response to genotoxicity induced by irradiation, cytotoxic drugs, and carcinogens through mediating cell cycle arrest and induction of apoptosis.^7^ p53 is implicated in a wide array of cellular activities by forming complex signaling networks with various molecular pathway members, such as ER.^8^ Somatic mutation in the p53 gene occurs frequently in human malignant neoplasms, including breast cancer. The p53-wildtype protein has a short half-life with a low level of intracellular accumulation. Stabilization of p53 protein without a stimulus, such as DNA damage, is associated with the loss of function secondary to a mutation or interaction with a viral or cellular oncoprotein.^9^ A meta-analysis^10,11^ revealed an association between p53 alterations (overexpression and mutations) and poor overall survival (OS) but not recurrence-free survival (RFS) in breast cancer. Similar results were obtained in the analysis of approximately 10 000 patients with breast cancer at the cBioPortal for Cancer Genomics.^12^

A patient- or tumor-related prognostic factor is associated with clinical outcome in the absence of therapy and reflects the natural history of a disease.^13^ However, assessment of prognostic factors has been confounded by treatments, and principles and methods related to the evaluation of prognostic factors are not well established.^12^ It is unclear why RFS is sometimes but not always concordant with OS and the performance of an individual prognostic factor in a disease state varies frequently in different studies.^14,15^ We postulated that function of molecular and clinical prognostic factors is affected by specific treatments and may vary owing to the nature of clinical outcomes. The hypothesis was tested through identification of independent prognostic variables for OS and RFS by homogeneous treatment modality in contrast to no treatment using multivariable Cox proportional hazards regression models. We also evaluated OS and RFS in women with p53-positive vs p53-negative tumors undergoing monotherapy after diagnosis by Kaplan-Meier analysis.

## Methods

### Study Population and Molecular Measurements

Patients were diagnosed with invasive breast cancer from 1985 to 1997 in the hospital centers of 4 geographical regions of the United States and participated in the accreditation program of the Commission on Cancer of the American College of Surgeons.^16,17^ The Breast Cancer Tissue Project received full review and approval by the institutional review board at each participating site.^16^ The collection of surgical specimens, unless the frozen samples needed to be collected, was performed with waiver of informed consent as appropriate. The established data set was coded and centrally maintained and contains age at diagnosis, clinicopathological variables, types of treatment received, and vital and recurrence status with a maximum of 282 months (23.5 years) of follow-up. Treatments included chemotherapy, endocrine therapy, radiotherapy, or other type of therapy in addition to surgery. The present study of deidentified human tumor specimens and data set was granted exempt status by the Office of Human Research Protections, National Institutes of Health, Bethesda, Maryland. The report adheres to the Transparent Reporting of a Multivariable Prediction Model for Individual Prognosis or Diagnosis (TRIPOD) reporting guideline for diagnostic/prognostic study and the REMARK reporting recommendations for tumor marker prognostic studies.^18^

Estrogen receptor, PR, and *ERBB2* status were centrally assayed and evaluated by pathologists from the Cooperative Breast Cancer Tissue Resource according to the American Society of Clinical Oncology and the College of American Pathologists guidelines.^19,20^ Expression of p53 protein was examined on formalin-fixed paraffin-embedded primary tumors in tissue microarray established from the tumor blocks of breast cancer specimens by immunohistochemistry with the use of DO7 antibody.^17,21^ p53 staining in 10% or more of the malignant nuclei was prespecified as p53 positive.

### Statistical Analysis

Data were analyzed from June 10, 2019, to March 18, 2020. The length of follow-up for OS was defined as the number of months from the date of diagnosis to the date of death due to any cause or to the date last known alive. The length of RFS was calculated as the number of months from the date of diagnosis to the date of first occurrence of ipsilateral breast tumor recurrence, locoregional recurrence (chest wall and ipsilateral axillary and internal mammary node areas), distant recurrence, or death due to any cause. Two cases with unavailable recurrence information were excluded from the RFS analysis in the endocrine treatment group, 6 in the no-treatment group, 5 in the chemotherapy group, and 5 in the combination treatment group. The primary analysis used the Cox proportional hazards regression model incorporating age at diagnosis (with 50 years as the cut point), tumor grades 2 and 3, ER status, *ERBB2* status, and p53 status as categorical variables and tumor size and number of positive nodes as continuous variables in distinct monotherapy groups to identify independent prognostic factors for OS and RFS. The Cox proportional hazards regression model was also used to estimate the risk of death by age groups younger than 40, 40 to 49, 50 to 59, 60 to 69, and 70 years or older in untreated patients. A likelihood ratio test estimated the performance of molecular and clinical variables in association with OS and RFS with corresponding 95% CIs. The event numbers for OS and RFS were 75 and 60, respectively, for the endocrine therapy group; 127 and 93, respectively, for the no-treatment group; 34 and 31, respectively, for the radiotherapy group; and 68 and 57, respectively, for the chemotherapy group. Based on a general rule of statistics of using 15 events (such as death or recurrence) per variable for time-to-event end point, each treatment group had adequate statistical power for the identification of at least 2 independent prognostic variables for OS or RFS.^22^ The secondary objective was to compare OS and RFS between p53-positive and p53-negative patients undergoing uniform therapy as well as those without treatment by Kaplan-Meier analysis. The differences in OS and RFS between p53-positive and p53-negative groups were compared by log-rank test. A χ^2^ test of association was used to compare categorical variables between p53-positive and p53-negative tumors. All statistical tests were 2 sided, and the significance level was prespecified at *P* = .05. Statistical analyses were performed using Prism, version 7 (GraphPad) and Lifelines, version 0.24.1 (Python).

## Results

Of 956 patients included in the analysis, median age was 61 (range, 25-96) years, and median follow-up time for OS was 115.5 (range, 5.0-282.0) months. The median follow-up for RFS was 87.0 (range, 1.0-282.0) months. Among 785 patients with p53 expression ascertained, 227 had undergone surgery alone as the treatment of their disease without systemic treatment and locoregional radiotherapy (regarded as untreated or no therapy). Three hundred twenty-six patients received monotherapy, including 113 with chemotherapy, 130 with endocrine therapy, 82 with radiotherapy, and 1 with other treatment that was excluded from outcome analysis, and 232 underwent the combination therapy.

### p53 Expression

Among primary tumors analyzed for p53 expression, we obtained p53 measurement for 785 cases. Overexpression of nuclear p53 protein was detected in 177 individuals (22.5%) with invasive breast cancer (eFigure 1 in the Supplement). The accumulation of nuclear p53 was significantly associated with younger age at diagnosis (70 of 177 [39.5%] vs 138 of 608 [22.7%] younger than 50 years; *P* < .001) and aggressive tumor features such as grade 3 tumors (112 of 177 [63.3%] vs 134 of 608 [22.0%]; *P* < .001) and more *ERBB2* positivity (50 of 177 [28.2%] vs 72 of 608 [11.8%]; *P* < .001). In addition, there were 69 of 177 ER-positive tumors (39.0%) and 71 of 177 PR-positive tumors (40.1%) in p53-positive cases vs 478 of 608 ER-positive tumors (78.6%) and 423 of 608 PR-positive tumors (69.6%) in p53-negative cases (*P* < .001) (eTable in the Supplement).

### Clinical Measures and Outcomes Without Treatments

**Figure 1.**
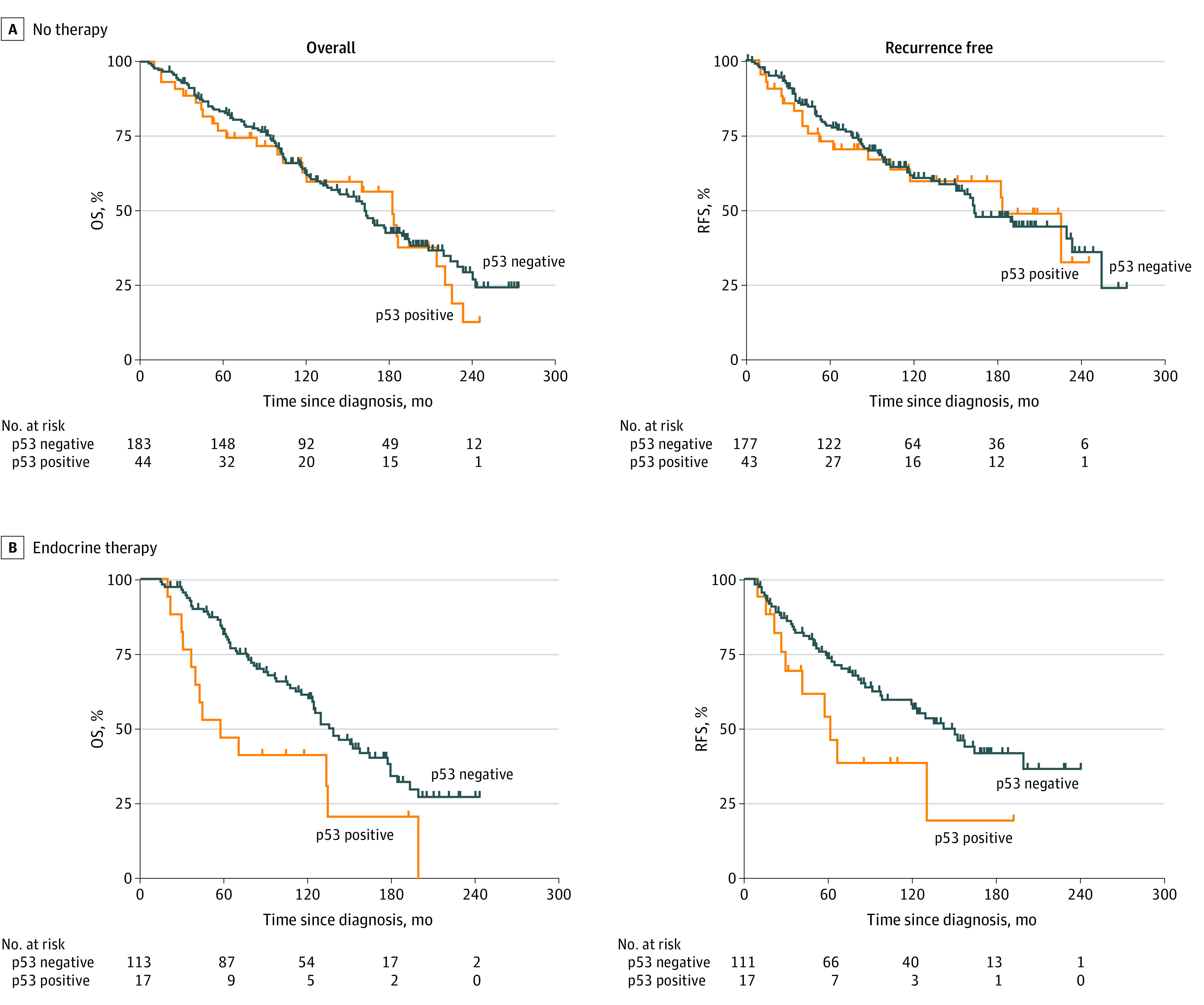
Association of p53 Overexpression With Overall Survival (OS) and Recurrence-Free Survival (RFS) in Patients With Breast Cancer The whiskers on the Kaplan-Meier survival plots represent the censored patients. For patients receiving no therapy, p53 positivity was not associated with worse OS (26 of 44 [59.1%] vs 101 of 183 [55.2%]; *P* = .60) or RFS (18 of 43 [41.9%] vs 75 of 177 [42.4%]; *P* = .92). For patients receiving endocrine therapy, p53 positivity was significantly associated with worse OS (13 of 17 [76.5%] vs 62 of 113 [54.9%]; *P* = .01) and RFS (10 of 17 [58.8%] vs 49 of 111 [44.1%]; *P* = .04).

**Figure 2.**
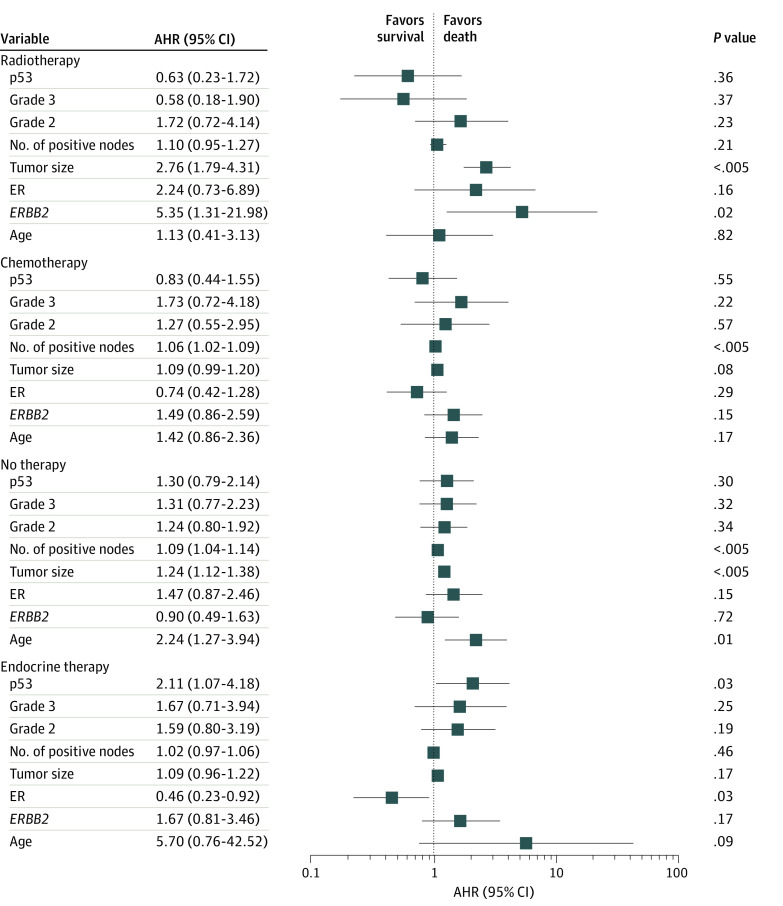
Risk of Death in Patients With Homogeneous and No Therapy by Cox Proportional Hazards Regression Analysis Adjusted hazard ratio (AHR) of 1.00 indicates lack of association; greater than 1.00, an increased risk of death; and less than 1.00, a decreased risk of death in the forest plot. Error bars indicate 95% CI. ER indicates estrogen receptor.

**Figure 3.**
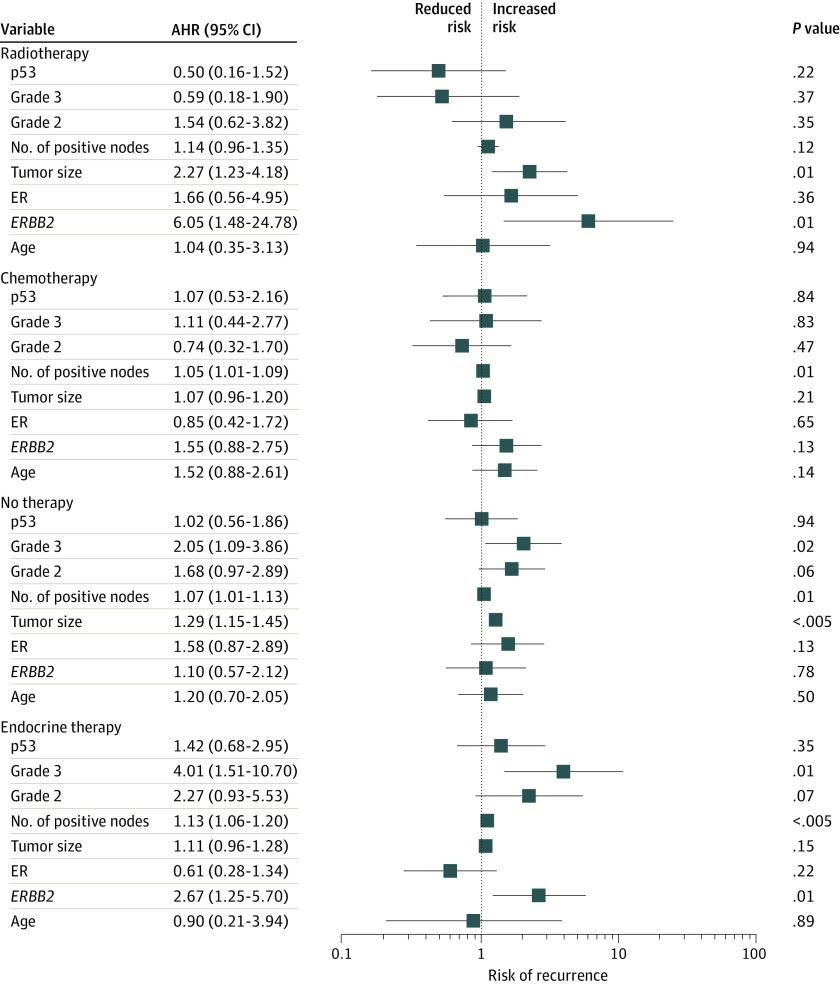
Risk of Recurrence in Patients With Homogeneous Therapy and No Therapy by Cox Proportional Hazards Regression Analysis Adjusted hazard ratio (AHR) of 1.00 indicates lack of association; greater than 1.00, an increased risk of recurrence; and less than 1.00, a decreased risk of recurrence in the forest plot. Error bars indicate 95% CI. ER indicates estrogen receptor.

No significant difference was observed between patients with p53-positive and p53-negative tumors without treatment in OS (26 of 44 [59.1%] vs 101 of 183 [55.2%]; *P* = .60) and RFS (18 of 43 [41.9%] vs 75 of 177 [42.4%]; *P* = .92) by Kaplan-Meier analysis (Figure 1A). In multivariable Cox proportional hazards regression models, older age (adjusted hazard ratio [AHR], 2.24; 95% CI, 1.27-3.94; *P* = .01) was significantly associated with poor OS (Figure 2). High grade (AHR, 2.05; 95% CI, 1.09-3.86; *P* = .02) instead of age was significantly associated with inferior RFS (Figure 3). As expected, larger tumor size (AHR for OS, 1.24 [95% CI, 1.12-1.38; *P* < .005]; AHR for RFS, 1.29 [95% CI, 1.15-1.45; *P* < .005]) and number of positive nodes (AHR for OS, 1.09 [95% CI, 1.04-1.14; *P* < .005]; AHR for RFS, 1.07 [95% CI, 1.01-1.13; *P* = .01]) were independent clinical measurements for both outcomes (Figure 2 and Figure 3).

### Clinical Measures and Outcomes by Endocrine Therapy

Kaplan-Meier analysis revealed that compared with p53-negative tumors, p53 positivity was significantly associated with worse OS (13 of 17 [76.5%] vs 62 of 113 [54.9%]; *P* = .01) and RFS (10 of 17 [58.8%] vs 49 of 111 [44.1%]; *P* = .04). The association of p53 with endocrine therapy outcomes was long-lasting throughout follow-up (Figure 1B). In the multivariable proportional hazards regression model, AHR of mortality for p53 status was 2.11 (95% CI, 1.07-4.18; *P* = .03) and for ER status was 0.46 (95% CI, 0.23-0.92; *P* = .03) (Figure 2). In contrast, the number of positive nodes (AHR, 1.13; 95% CI, 1.06-1.20; *P* < .005), high grade (AHR, 4.01; 95% CI, 1.51-10.70; *P* = .01), and *ERBB2* positivity (AHR, 2.67; 95% CI, 1.25-5.70; *P* = .01) were significantly associated with higher risk of recurrence (Figure 3). Notably, although p53 and ER were independent indicators of survival, the number of positive nodes, high tumor grade, and *ERBB2* were significantly associated with the recurrence outcome independent of other clinical parameters after endocrine therapy alone.

### Clinical Measures and Outcomes by Radiotherapy and Chemotherapy

During the long-term follow-up, p53 status was not significantly associated with OS (7 of 18 [38.9%] p53-positive vs 27 of 64 [42.2%] p53-negative; *P* = .92) and RFS (4 of 17 [23.5%] p53-positive vs 27 of 64 [42.2%] p53-negative; *P* = .32) for radiotherapy or with OS (21 of 38 [55.3%] p53-positive vs 47 of 75 [62.7%] p53-negative; *P* = .89) and RFS (22 of 37 [59.5%] p53-positive vs 35 of 70 [50.0%] p53-negative; *P* = .33) for chemotherapy by Kaplan-Meier analysis (eFigure 2 in the Supplement). Multivariable Cox analysis demonstrated a significant association after radiotherapy with inferior OS for larger tumors (AHR, 2.76, 95% CI, 1.79-4.31; *P* < .005) and *ERBB2* (AHR, 5.35; 95% CI, 1.31-21.98; *P* = .02) and with RFS for larger tumors (AHR, 2.27; 95% CI, 1.23-4.18; *P* = .01) and *ERBB2* (AHR, 6.05; 95% CI, 1.48-24.78; *P* = .01) (Figure 2 and Figure 3). The number of positive nodes was significantly relevant to chemotherapy-associated OS (AHR, 1.06; 95% CI, 1.02-1.09; *P* < .005) and RFS (AHR, 1.05; 95% CI, 1.01-1.09; *P* = .01) independent of other clinical and molecular factors in the Cox models (Figure 2 and Figure 3).

## Discussion

We systematically evaluated the outcome of various homogeneous therapies associated with reference to nontreatment within a patient population. Our results demonstrated a substantial variation in the identification of independent prognostic factors for OS and RFS, which was weighted by treatment modality and outcome type. Age was identified as an independent poor prognostic factor for OS vs high grade for RFS in untreated patients, in addition to the tumor size and number of positive axillary lymph nodes for both outcomes.^23^ After dividing the patients into multiple age groups, we observed an increased risk of mortality by increasing age from 40 to 49 years to 50 to 59 years, 60 to 69 years, and 70 years or older, except those who were younger than 40 years, by univariate Cox proportional hazards regression analysis (eFigure 3 in the Supplement). Overall, according to the National Cancer Institute’s Surveillance, Epidemiology, and End Results Statistics, survival rates for breast cancer decrease as age increases. Similarly, increasing breast cancer mortality is associated with older age according to the cancer statistics from the American Cancer Society.^24^ As stated, the outcomes of untreated patients reflect the natural history of breast cancer, and these factors are bona fide prognostic factors in patients with breast cancer after diagnosis.^25^

Estrogen receptor positivity demonstrated an independent power for better prognosis in patients who received endocrine therapy alone by multivariable Cox proportional hazards regression analysis. Comparatively, it did not reach statistical significance in other monotherapy groups and the nontreatment group. The data indicate that the role of ER in favorable prognosis was largely ascribed to the endocrine therapy, relative to other types of treatment and nontherapy. In a Surveillance, Epidemiology, and End Results population-based study with a mix of treatments, the association between ER and survival prognosis was nonproportional over time.^26^ That is, patients with ER-positive tumors had better survival in early years after diagnosis, and the survival improved for individuals with ER-negative tumors at and after 7 years, because of constant ER-positive mortality hazard rates and decreasing ER-negative hazard rates after peaking at 17 months. A substantial decrease in the survival rate within 5 years had been observed in treated vs untreated patients with triple-negative breast cancer; in contrast, a low but steady decrease of survival has been observed in patients with HR-positive and *ERBB2*-negative breast cancer.^27^ Noticeably, the tumor size no longer had an independent role after endocrine therapy compared with nontreatment, consistent with the previous report in women who were treated with endocrine therapy alone in National Surgical Adjuvant Breast and Bowel Program trials.^28^

Herein, we also provided evidence that overexpression of p53 was significantly associated with poor survival after endocrine therapy. Notably, p53, in addition to ER, exerted more weight on OS than any other clinical parameters such as age, number of positive nodes, tumor size, and grades. Modulation of ER by tamoxifen or fulvestrant led to unleashing of p53 with either normal or aberrant activity from the ER-p53 complex in which ER represses p53’s transactivation function.^8,29^ Such treatments resulted in better outcomes in patients with ER-positive tumors that express wildtype than mutant p53. In addition, *TP53* mutation not only was involved in the de novo resistance in primary tumors but was also associated with poor survival in HR-positive and *ERBB2*-negative metastatic breast cancer.^30^ The alteration also correlated with the resistance to other endocrine agents such as palpociclib (*r* = −0.992; *P* < .001) and raloxifene hydrochloride (*r* = −0.994; *P* < .001) in a panel of breast cancer cell lines by data analysis using CellMiner, version 2.2 (https://discover.nci.nih.gov/cellminer/). The data may be critical to an approach of precision endocrine therapy in the care of patients with breast cancer.^31^ Our results, other real-world data, and clinical trials are gathering sufficient evidence for the cancer research community and regulatory agencies to consider exclusion of p53-positive and HR-positive breast cancer from endocrine therapy or to use alternative treatment approaches.^8,29,32,33,34,35^ In current practice after TAILORx (Trial Assigning Individualized Options for Treatment) trial results, approximately 70% of patients with HR-positive and *ERBB2*-negative early-stage breast cancer receive endocrine therapy alone, which accounts for as much as 50% of all early-stage breast cancers.^36^

Clinical measurements (nodal status, high grade, and *ERBB2*) that weighted independently for RFS were different from the survival factors in the case of endocrine therapy. This type of discordance between survival and recurrence or progression outcomes was also described in other treatment circumstances, such as in the treatment of advanced solid tumors by programmed cell death 1–blocking antibodies by a meta-analysis.^37^

As for locoregional radiotherapy alone, *ERBB2* positivity and larger tumor size were identified as the independent prognosticators of both inferior survival and recurrence outcomes. A systematic review and meta-analysis revealed that the rate of locoregional control was worse in patients with *ERBB2*-positive tumors than luminal A tumors in breast cancer.^38^ As expected, tumor size was inversely associated with OS and RFS from the locoregional management.

Significantly, the number of positive nodes had the greatest value among the molecular and clinical measurements after chemotherapy and was an independent prognostic factor for OS and RFS in the Cox multivariable proportional hazards regression models. Age, larger tumor size, and *ERBB2* positivity demonstrated nonsignificant trends toward poor chemotherapy outcomes. The data were in agreement with other chemotherapy data.^36,39,40^

### Strengths and Limitations

Strengths of this study include the novel connection of the performance of prognostic variables to distinct therapy and demonstrating their differential and nondifferential association with OS and RFS by treatment modality relative to nontreatment. The untreated patients were analyzed as an independent entity in the evaluation of clinical and molecular factors for bona fide prognosis. Each homogeneous treatment group, with a long-term follow-up, had adequate statistical power for the identification of at least 2 independent prognostic factors for OS and RFS, respectively. This study was a population-based cohort study, with well-organized and high-quality molecular and clinical data. Limitations include a lack of randomization; however, current practice does not allow a group without treatment (except node-negative breast tumor that is 0.5 cm or smaller) and/or homogeneous therapy in patients with certain patient and tumor characteristics.

## Conclusions

In this study, prognostic factors were associated with specific treatment and weighted by the outcome category with reference to untreated patients. Thus, the clinical and molecular measurements in the context of treatment should be regarded as the treatment-associated prognostic factors for OS and/or RFS. We anticipate that the knowledge derived from this study could set a basis to pinpoint independent prognostic factors related to a treatment modality and provide clarity for the evaluation of surrogate markers for OS. These findings shed light on the precision assessment of clinical prognostic tools in the management of breast cancer and perhaps in other diseases.

## Notes

Supplement. **eFigure 1.** Nuclear Expression of p53 Protein in Primary Breast Tumors **eFigure 2.** Effects of p53 Expression on OS and RFS in Patients With Breast Cancer **eFigure 3.** Evaluation of Risk of Mortality by Dividing Untreated Patients Into Multiple Age Groups **eTable.** p53 Status in Association With Patient and Clinicopathologic Factors in Breast Cancer
