# Supplementary material for: Association of Independent Prognostic Factors and Treatment Modality With Survival and Recurrence Outcomes in Breast Cancer
Source: JAMA Netw Open. 2020 Jul 9;3(7):e207213. doi: 10.1001/jamanetworkopen.2020.7213 (PMC7348688; doi:10.1001/jamanetworkopen.2020.7213)
Supplement: Supplement. — eFigure 1. Nuclear Expression of p53 Protein in Primary Breast Tumors eFigure 2. Effects of p53 Expression on OS and RFS in Patients With Breast Cancer eFigure 3. Evaluation of Risk of Mortality by Dividing Untreated Patients Into Multiple Age Groups eTable. p53 Status in Association With Patient and Clinicopathologic Factors in Breast Cancer [file jamanetwopen-3-e207213-s001.pdf]

Supplementary Online Content

Nguyen D, Yu J, Reinhold WC, Yang SX. Association of independent prognostic factors and treatment modality with survival and recurrence outcomes in breast cancer. *JAMA Netw Open*. 2020;3(6):e207213. doi:10.1001/jamanetworkopen.2020.7213

eFigure 1. Nuclear Expression of p53 Protein in Primary Breast Tumors
eFigure 2. Effects of p53 Expression on OS and RFS in Patients With Breast Cancer
eFigure 3. Evaluation of Risk of Mortality by Dividing Untreated Patients Into Multiple Age Groups
eTable. p53 Status in Association With Patient and Clinicopathologic Factors in Breast Cancer

This supplementary material has been provided by the authors to give readers additional information about their work.

©2020 Nguyen D et al. *JAMA Network Open*.

eFigure 1. Nuclear Expression of p53 Protein in Primary Breast Tumors

Shown are the representative staining of p53-negative tumors (A1 and A2) and p53-positive tumors (B1 and B2). Note that the two p53-positive tumors were derived from patients with high-grade triple-negative breast cancer. Original magnification, x600; scale bar, 50 µm.

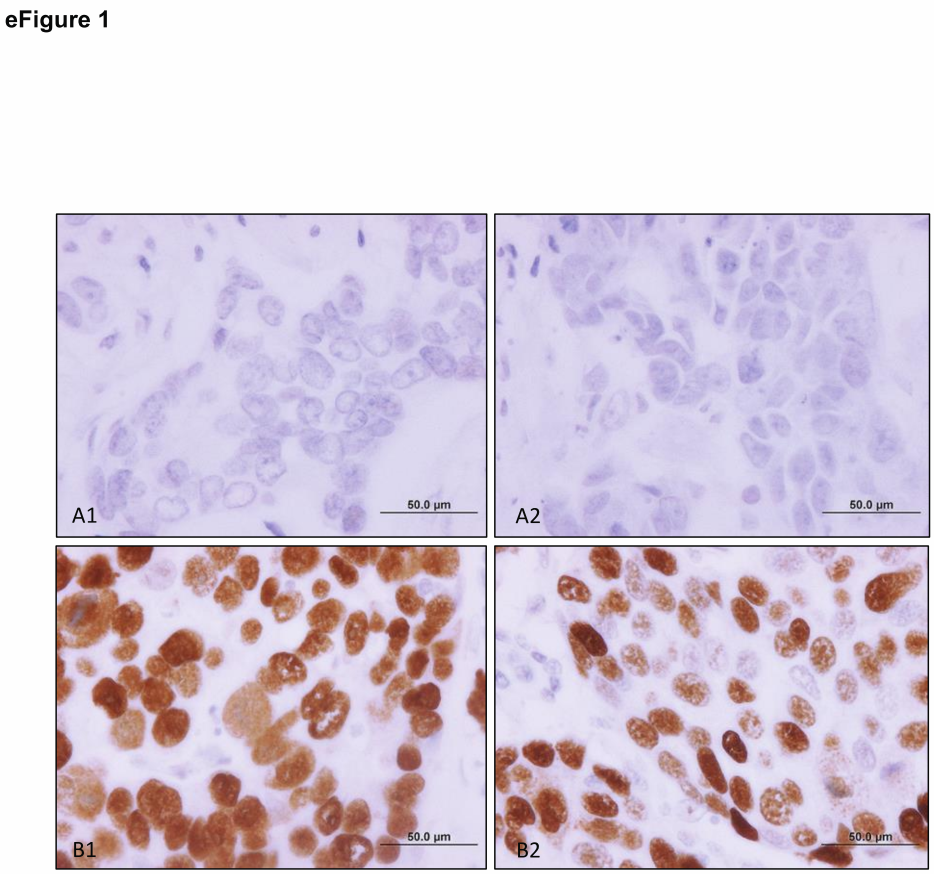

©2020 Nguyen D et al. *JAMA Network Open*.

eFigure 2. Effects of p53 Expression on OS and RFS in Patients With Breast Cancer

OS and RFS in patients treated with radiotherapy alone (A and B), chemotherapy alone (C and D), and combination therapy (E and F). Numbers and death or recurrence rates in red indicate p53-positive cases. The whiskers on the Kaplan-Meier survival plots represent the censored subjects. OS, overall survival; RFS, recurrence-free survival.

eFigure 2

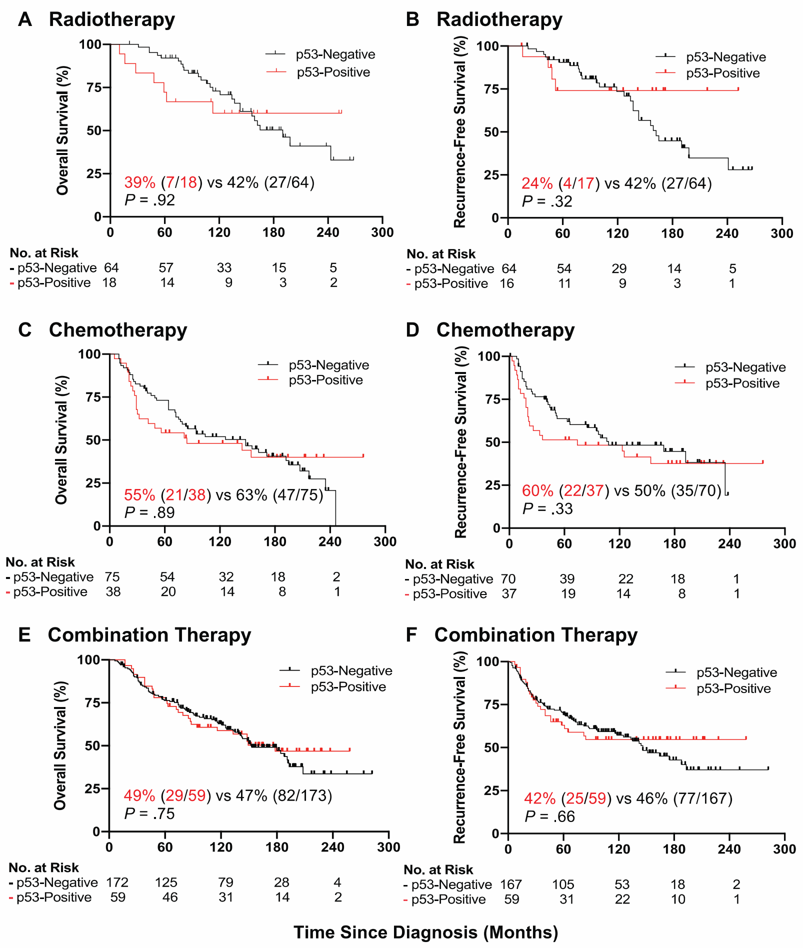

©2020 Nguyen D et al. *JAMA Network Open*.

eFigure 3. Evaluation of Risk of Mortality by Dividing Untreated Patients Into Multiple Age Groups

The age groups are shown in the x-axis and the dotted line indicates the hazard ratio of 1.

eFigure 3

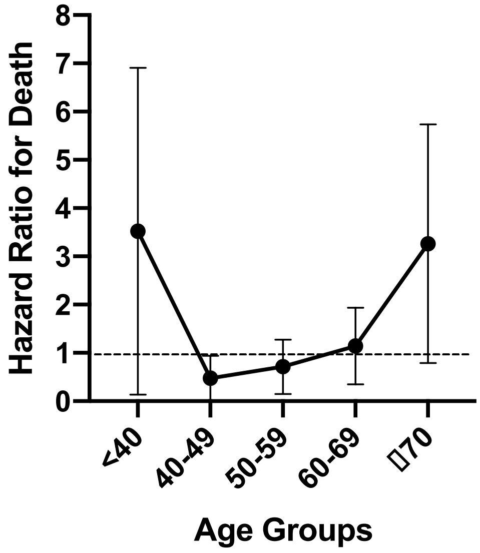

©2020 Nguyen D et al. *JAMA Network Open*.

eTable. p53 Status in Association With Patient and Clinicopathologic Factors in Breast Cancer

Variable	Total (n=785) No. (%)	p53-Negative (n=608) No. (%)	p53-Positive (n=177) No. (%)	*P* value
Age at diagnosis				<.001
< 50	208 (26.5)	138 (22.7)	70 (39.5)	
≥ 50	577 (73.5)	470 (77.3)	107 (60.5)	
Histology				.003
Ductal	727 (92.6)	554 (91.1)	173 (97.7)	
Lobular	58 (7.4)	54 (8.9)	4 (2.3)	
T stage				.073
T1	436 (55.5)	351 (57.7)	85 (48.0)	
T2	216 (27.5)	160 (26.3)	56 (31.6)	
T3	85 (10.8)	59 (9.7)	26 (14.7)	
T4	48 (6.1)	38 (6.3)	10 (5.7)	
N stage				.095
N0	431 (54.9)	343 (56.4)	88 (49.7)	
N1	304 (38.7)	226 (37.2)	78 (44.1)	
N2	49 (6.2)	39 (6.4)	10 (5.6)	
N3	1 (0.1)	0 (0.00)	1 (0.6)	
Tumor size				.071
≤ 2 cm	437 (55.7)	352 (57.9)	85 (48.0)	
> 2 to ≤ 5 cm	246 (31.3)	183 (30.1)	63 (35.6)	
> 5 cm	101 (13.0)	73 (12.0)	28 (15.8)	
Missing	1		1 (0.6)	
Tumor grade				<.001
I	180 (22.9)	171 (28.1)	9 (5.1)	
II	359 (45.7)	303 (49.8)	56 (31.6)	
III	246 (31.3)	134 (22.0)	112 (63.3)	
ER status				<.001
Negative	196 (25.0)	97 (16.0)	99 (55.9)	
Positive	547 (69.7)	478 (78.6)	69 (39.0)	
Missing	42 (5.3)	33 (5.4)	9 (5.1)	
PR status				<.001
Negative	250 (31.9)	152 (25.0)	98 (55.4)	
Positive	494 (62.9)	423 (69.6)	71 (40.1)	
Missing	41 (5.2)	33 (5.4)	8 (4.5)	
HER2 status				<.001
Negative	628 (80.0)	508 (83.6)	120 (67.8)	
Positive	122 (15.5)	72 (11.8)	50 (28.2)	
Missing	35 (4.5)	28 (4.6)	7 (4.0)	
Triple-negative status				<.001
No	637 (81.1)	524 (86.2)	113 (63.8)	
Yes	109 (13.9)	52 (8.6)	57 (32.2)	
Missing	39 (5.0)	32 (5.2)	7 (4.0)	
Adjuvant therapy				.001
None	227 (28.9)	183 (30.1)	44 (24.9)	
Endocrine therapy	130 (16.6)	113 (18.6)	17 (9.6)	
Radiotherapy	82 (10.5)	64 (10.5)	18 (10.2)	
Chemotherapy	113 (14.4)	76 (12.5)	38 (21.5)	
Other therapy	1 (0.10)	0 (0.00)	1 (0.5)	
Combination	232 (29.5)	172 (28.3)	59 (33.3)	

Abbreviations: ER, estrogen receptor; HER2, human epidermal growth receptor 2; n, number; PR, progesterone receptor.

©2020 Nguyen D et al. *JAMA Network Open*.
